# An antibiotic stewardship initiative focused on community-acquired bacterial pneumonia (CABP) in outpatient clinics and urgent care centers: a 2023–2024 community health system experience

**DOI:** 10.1017/ash.2025.10100

**Published:** 2025-08-15

**Authors:** Tomefa E. Asempa, Tyler Ackley, Kristin E. Linder, Cara D. Riddle, Eric Walsh, David P. Nicolau

**Affiliations:** 1 Center for Anti-Infective Research and Development, Hartford Hospital, Hartford, CT, USA; 2 Pharmacy Department, Hartford Hospital, Hartford, CT, USA; 3 GoHealth Urgent Care, Hartford, CT, USA; 4 Hartford Healthcare Medical Group, Farmington, CT, USA; 5 Division of Infectious Diseases, Hartford Hospital, Hartford, CT, USA

## Abstract

**Objective:**

This before–after study aimed to evaluate whether an order-set intervention would improve CABP-guideline concordance among outpatients.

**Setting:**

This study included adult patients presenting to outpatient clinics (*n* = 92) and urgent care centers (*n* = 39) within a community-based health system without a formal outpatient antibiotic stewardship program (ASP).

**Intervention:**

The intervention consisted of an antibiotic order-set and awareness campaign. Patient encounters were identified via CABP ICD-10 codes and IDSA-relevant patient comorbidities (chronic heart, lung, liver, or renal disease; diabetes mellitus; alcoholism; malignancy; asplenia) were extracted from the electronic health record. Primary outcome was to describe the proportion of patients receiving concordant therapy per IDSA guideline and local antibiogram in a pre- (May 2023 – April 2024) and post-intervention period (May 2024 – December 2024).

**Results:**

Baseline and intervention antibiotic concordance rate was 33.3% (1,467/4,401 encounters) and 28.0% (1,388/4,954 encounters), respectively. Among patients with no comorbidity, monotherapy prescriptions (concordant and discordant) decreased post-intervention and were replaced by higher levels of combination therapy (15% increase), albeit all discordant due to lack of comorbidities. Among patients with comorbidities, combination antibiotics increased by 12% post-intervention, driven by concordant prescriptions including amoxicillin/clavulanate plus azithromycin while the most frequently prescribed discordant combination was amoxicillin plus azithromycin. Trends were similar in primary care and urgent care centers.

**Conclusions:**

A stewardship intervention, including an order-set and awareness campaign improved the selection of combination therapy for appropriate patients but did not improve overall guideline concordance. For health systems without a dedicated outpatient ASP, these data will help bolster stewardship efforts towards more effective strategies.

## Introduction

Inappropriate antibiotic use contributes to antibiotic resistance and is associated with antibiotic-related adverse events such as allergies and *Clostridioides difficile* infection.^1–3^ Antimicrobial stewardship programs (ASPs) have been instituted inpatient within many health systems to address this issue and optimize antibiotic use.^2,4,5^ However, the benefit of ASPs in optimizing antibiotic prescriptions in the outpatient setting, including urgent care centers—which is one of the fastest growing settings for outpatient care in the United States, remains unclear.^2,6^

Most antibiotics prescribed to outpatients are for the treatment of acute respiratory infections including bronchitis and upper respiratory tract infections.^7^ However, approximately 1 in 2 outpatient antibiotic prescriptions might be inappropriate.^1,8–10^ Given this data, outpatient stewardship interventions have been focused on reducing antibiotic prescriptions, however there is limited evidence assessing optimal antibiotic selection in the outpatient setting with respect to current guidelines.^11^

The Centers for Disease Control and Prevention (CDC) and The Joint Commission have published core elements of outpatient antibiotic stewardship to provide a framework for improving antibiotic prescribing among outpatient clinicians that are focused on commitment, policy, data tracking and reporting, as well as education.^8,12^ However, it is the clinical practice guidelines, including the 2019 American Thoracic Society and Infectious Diseases Society of America community-acquired pneumonia (CAP) guideline, that provides evidence-based data and guidance for clinicians on diagnostic and therapeutic decisions when managing outpatients.^13,14^

Here we describe the implementation and results of a quality improvement stewardship intervention to improve guideline-concordant antibiotic prescribing for community-acquired bacterial pneumonia (CABP) in primary care clinics and urgent care centers.

## Methods

The study was approved by the Hartford Hospital institutional review board and deemed exempt via a waiver (HHC-2022-0220).

### Study design and population

Hartford HealthCare includes the flagship Hartford Hospital and 6 community hospitals, 92 primary care clinics, and 39 GoHealth affiliated urgent care centers throughout Connecticut. Each clinic is staffed by physicians and advanced level practitioners. The inpatient ASP across Hartford HealthCare was initiated in 2014 and is led by 4.0 full-time equivalent (FTE) Infectious Diseases (ID) pharmacists and supported by 0.8 FTE ID physicians. However, within this community-based health system, no formal outpatient or ambulatory stewardship program exists thus the implementation of an electronic order set to guide prescribers on CABP-concordant antibiotic selection was considered a reasonable approach.

A pre-post intervention study design was implemented to assess the impact of the stewardship intervention across primary care clinics and urgent care centers. The prespecified dates compared a baseline period (preintervention: May 2023–April 2024) with an intervention window (May 2024–December 2024).

### Intervention

Our intervention was based on (a) integration of an antibiotic order set in the electronic health record that highlighted guideline-concordant antibiotic recommendations according to patient comorbidities (Supplemental). (b) order-set awareness campaign at departmental huddle meetings pre and postlaunch, and (c) 2-minute video provided to all providers by department heads that narrates and demonstrates how to access the order set and place an antibiotic order.

The order set was created to seamlessly integrate with current prescriber workflow and was accessed with a synonym search strategy of key words including: “CAP,” “Community Acquired Pneumonia,” “Amoxicillin,” “Amox/Clav,” “Augmentin,” “Amoxil,” “Doxycycline,” “Vibramycin,” “Azithromycin,” “Z-Pak,” “Zithromax,” “ZPAK,” “Z Pak,” “ZPak,” “Cefuroxime,” “Ceftin,” “Levofloxacin,” or “Levaquin.” Furthermore, the use of azithromycin monotherapy was cautioned based on local microbiological resistance rates demonstrating that *Streptococcus pneumoniae* resistance to macrolide exceeded 25% across Hartford Healthcare System emergency departments in 2023 (antibiogram data on file).

### Study outcome

Data were electronically extracted from the EHR for each adult patient (18 yr or older) encounter based on the following *International Classification of Diseases, Tenth Revision, Clinical Modification* (*ICD-10-CM*) CABP-related codes: “A48.1,” “A49.0,” “A49.01,” “A49.1,” “A49.3,” “B95.0,” “B95.3,” “B95.4,” “B95.5,” ”B96.0,” “B96.1,” “B96.3,” “J13,” “J14,” “J15,” “J16.0,” “J18.0,” “J18.1,” “J18.8,” and “J18.9”; Supplemental. Data extracted from each medical record included patient demographic characteristics, encounter location, date and time, payor type, and antibiotic prescriptions ordered. IDSA relevant patient comorbidities (chronic heart, lung, liver, or renal disease; diabetes mellitus; alcoholism; malignancy; asplenia) were extracted from patient problem lists. Patients with an ordered antibiotic in the 30 days preceding index visit that is, washout period were excluded. A random selection of cases (1 in 10 encounters) during baseline and intervention time frames was reviewed for pneumonia diagnosis and antibiotic prescription accuracy.

The primary outcome was to describe the proportion of patients receiving concordant therapy (as defined by antibiotic choice and prescription of monotherapy or combination therapy depending on presence of comorbidity, Supplemental) per IDSA guideline and local antibiogram.

### Statistical analyses

Descriptive statistics were performed using GraphPad Prism (version 10.4.1; Boston, Massachusetts USA). Any difference in the CABP-guideline discordance or concordance rates among patients with and without comorbidities was determined using the *χ*^2^ test, and a prespecified alpha level of .05. For additional analysis, concordant rates were also assessed based on patient encounter location (primary care clinics or urgent care centers).

## Results

A total of 4,401 and 4,954 patient encounters were identified in the baseline and intervention period, respectively (Table 1). Patient were predominantly female, white, and utilized commercial insurance. Patients were younger (age (mean [SD], 48 [18] vs 57 [18] years) in the intervention period and had fewer comorbidities (42.3% vs 59.8%). The most common clinical diagnosis code documented per encounter was J18.9 (pneumonia, unspecified organism; [baseline 90.7%; intervention 90.3%]).

**Table 1.** Patient demographics and characteristics among outpatient encounters by study period

Characteristics	Baseline (*N* = 4,401)	Intervention (*N* = 4,954)
**Age, mean (SD), y**	57 (18)	48 (18)
**Gender, *n* (%)**		
Female	2,582 (58.7%)	3,059 (61.7%)
Male	1,819 (41.3%)	1,895 (38.3%)
**Race, *n* (%)**		
American Indian or Alaska Native	10 (0.2%)	6 (0.1%)
Asian	92 (2.1%)	95 (1.9%)
Black or African American	194 (4.4%)	233 (4.7%)
Native Hawaiian or Other Pacific Islander	9 (0.2%)	6 (0.1%)
Other, declined or not provided	696 (15.8%)	931 (18.8%)
White	3,400 (77.3%)	3,683 (74.4%)
**Setting, *n* (%)**		
Outpatient clinic	1,687 (38.3%)	1,246 (25.2%)
Urgent care center	2,714 (61.7%)	3,708 (74.8%)
**Insurance, *n* (%)**		
Commercial	3,265 (74.2%)	3,827 (77.3%)
Government (Medicaid/Medicare)	1,061 (24.1%)	1,008 (20.3%)
Self-Pay or Other	75 (1.7%)	119 (2.4%)
**Comorbidity, *n* (%)**		
Present	2,633 (59.8%)	2,098 (42.3%)
Not present	1,768 (40.2%)	2,856 (57.7%)

**Figure 1.**
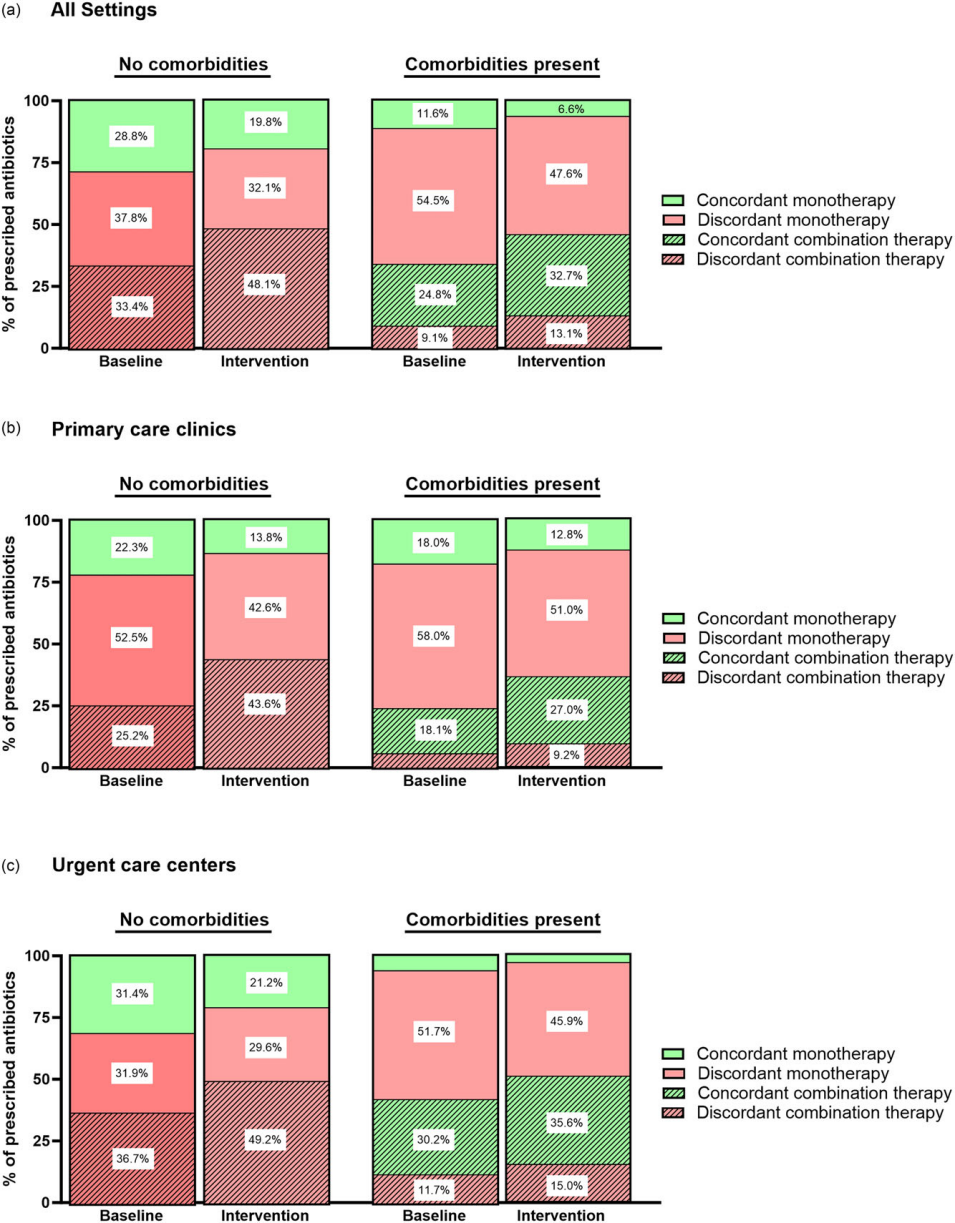
Proportion of concordant and discordant antibiotics per patient encounter during the baseline and intervention periods.

Overall, the guideline adherence based on antibiotic selection decreased from 33.3% during the baseline period to 28% in the intervention period. Table 2 details each concordance metric by antibiotic class and Figure 1 highlights the proportion of concordant and discordant monotherapies and combination therapies during the baseline and intervention periods.

**Table 2.** Guideline adherence of empirical antibacterial therapy in the study periods

Characteristics	Baseline	Intervention	*p*-value
All encounters	4,401	4,954	
**No comorbidities**	1,768 (40.2%)	2,856 (57.7%)	
Concordant monotherapy	509 (28.8%)	564 (19.8%)	<.05
Amoxicillin	139 (7.9%)	110 (3.9%)	
Doxycycline	370 (20.9%)	454 (15.9%)	
Discordant monotherapy	669 (37.8%)	917 (32.1%)	<.05
Amoxicillin/clavulanate	208 (11.7%)	212 (7.4%)	
Macrolide	283 (16.0%)	554 (19.4%)	
Others	178 (10.1%)	151 (5.3%)	
Discordant combination therapy	590 (33.4%)	1,375 (48.1%)	<.05
Amoxicillin/clavulanate + doxycycline	75 (4.2%)	139 (4.9%)	
Amoxicillin/clavulanate + macrolide	213 (12.1%)	515 (18.0%)	
Cephalosporin + doxycycline	12 (0.7%)	31 (1.1%)	
Cephalosporin + macrolide	44 (2.5%)	101 (3.5%)	
Others	246 (13.9%)	589 (20.6%)	
**With comorbidities**	2,633 (59.8%)	2,098 (42.3%)	
Concordant monotherapy	304 (11.6%)	138 (6.6%)	<.05
Respiratory fluoroquinolone	304 (11.6%)	138 (6.6%)	
Discordant monotherapy	1,435 (54.5%)	999 (47.6%)	<.05
Amoxicillin	100 (3.8%)	58 (2.8%)	
Amoxicillin/clavulanate	296 (11.2%)	173 (8.2%)	
Doxycycline	532 (20.2%)	336 16.0%)	
Macrolides	363 (13.8%)	360 (17.2%)	
Others	144 (5.5%)	72 (3.4%)	
Concordant combination therapy	654 (24.8%)	686 (32.7%)	<.05
Amoxicillin/clavulanate + doxycycline	177 (6.7%)	170 (8.1%)	
Amoxicillin/clavulanate + macrolide	330 (12.5%)	381 (18.2%)	
Cephalosporin + doxycycline	52 (2.0%)	44 (2.1%)	
Cephalosporin + macrolide	95 (3.6%)	91 (4.3%)	
Discordant combination therapy	240 (9.1%)	275 (13.1%)	<.05
Amoxicillin + macrolide	89 (3.4%)	193 (9.2%)	
Others	151 (5.7%)	82 (3.9%)	
Overall Concordance	1,467 (33.3%)	1,388 (28%)	<.05

### No comorbidities

Among patients with no comorbidity, both concordant (amoxicillin and doxycycline only) and discordant monotherapy prescriptions, decreased after the intervention while the proportion of patients receiving combination therapy (all discordant based on lack of patient comorbidities) increased by approximately 15% between the 2 periods. The most common discordant combination therapy prescribed in this patient cohort was amoxicillin/clavulanate plus azithromycin.

### Presence of comorbidities

The proportion of patients with comorbidities that received monotherapy decreased from 66% to 54% after the intervention. A large proportion of these orders were inappropriate that is doxycycline or azithromycin instead of a respiratory fluoroquinolone (Table 1). Antibiotic combination therapy increased by 12% after the intervention, driven largely by guideline-concordant prescriptions including amoxicillin/clavulanate plus azithromycin while the most frequently prescribed discordant combination was amoxicillin plus azithromycin.

### Encounter location

A greater proportion of patients visited the urgent care center (74.8%) during the intervention period compared with the baseline period (61.7%). The concordance rates and trends were similar across encounter locations. However, the increase in concordant combination therapy was more pronounced in primary care clinics.

## Discussion

We piloted an EHR-based stewardship program targeting antibiotic prescriptions for CABP across outpatient locations in a large community-based healthcare system. Baseline antibiotic concordance or appropriateness was low and consistent with reported data for upper respiratory tract infections, highlighting the room for improvement.^1,8–10^ Postintervention, we did not observe an increase in overall guideline concordance, however antibiotic selection among patients with comorbidities was improved and driven by an increase in appropriate combination therapy prescription. The use of fluoroquinolones, commonly associated with adverse events, was low overall and decreased further postintervention.

This intervention was the first antibiotic intervention conducted in the outpatient setting for this institution. Our findings highlight the challenge of outpatient stewardship efforts in community-based healthcare systems despite antibiotic prescribing rates that are similar to larger academic institutions.^15,16^ While our inpatient stewardship program is robust across 6 hospitals, with inpatient clinicians familiar with stewardship core metrics, the outpatient setting has not been a point of focus. With the growing outpatient and urgent care landscape, institutions will need to provide ASPs with resources to exceed The Joint Commission and CDC metrics.

The lack of improvement in overall concordance is likely multifactorial. Notably, successful stewardship interventions have a component of oversight and accountability such as audit and feedback as well as EHR justification prompts that require clinicians to provide a reason for discordant antibiotic orders.^17–20^ There have also been studies that have used achieving antibiotic stewardship goals as a part of clinician compensation. These accountability measures and financial incentives have been shown to have an influence on prescribing patterns.^16,21,22^ Inappropriate antibiotic prescribing in the management of upper respiratory illnesses is also high due to uncertainty regarding the etiology and the probability of viral, bacterial, or viral-bacterial coinfection. With this uncertainty is an increased frequency of inappropriate antibiotic prescriptions to please patients, improve satisfaction scores, and placate providers fear of misdiagnosis.^9,19,23,24^ Given the high severity of the 2024–2025 influenza season per the CDC, high antibiotic discordance including an increase in combination therapy during this study period is not entirely surprising.^25^ Despite the barriers discussed above, these data have spurred discussion among the stewardship team, healthcare leaders, and clinicians to put effort and resources into more robust data collection and interventions such as audit and feedback, continuous education, and involvement of ID-trained clinicians to improve patient care.

There are several limitations to consider. First, lack of a randomized control study design limits a determination of causality and also allows external factors such as an active influenza season to potentially affect the study intervention. Additionally, non-uniform intervention periods and lack of stratified sampling may have introduced unknown biases. We also performed a pre and-post analysis rather than an interrupted time-series analysis to assess prescribing patterns. Finally, patient outcome such emergency department visit and hospitalization was not assessed to ascertain the impact of antibiotic concordance. Of note, an important update in October 2024 was made during the study period whereby the Advisory Committee on Immunization Practices updated its vaccine recommendation, advising that all adults receive the pneumococcal vaccine starting at age 50, a shift from the previous age of 65 years. The uptake of this recommendation as well as the impact on disease burden and patient outcome has yet to be evaluated.^26^

## Conclusion

In summary, we present our collective experience of an antibiotic stewardship initiative targeting CABP that did not improve overall antibiotic prescribing patterns to align with 2019 CAP guidelines. A large proportion of patients (with and without comorbidities) received combination therapy, and azithromycin monotherapy orders were still elevated despite a caution regarding high *S. pneumoniae* resistance rates across the healthcare system. This study however provides valuable baseline data and opportunities to galvanize resources and optimize our stewardship efforts across the spectrum of care.

## Supporting information

10.1017/ash.2025.10100.sm001 Asempa et al. supplementary material Asempa et al. supplementary material

## Supplementary material

The supplementary material for this article can be found at https://doi.org/10.1017/ash.2025.10100.
