# Supplementary material for: An antibiotic stewardship initiative focused on community-acquired bacterial pneumonia (CABP) in outpatient clinics and urgent care centers: a 2023–2024 community health system experience
Source: Antimicrob Steward Healthc Epidemiol. 2025 Aug 15;5(1):e185. doi: 10.1017/ash.2025.10100 (PMC12394021; doi:10.1017/ash.2025.10100)
Supplement: Asempa et al. supplementary material [file S2732494X25101009sup001.docx]

**Supplemental**

**An Antibiotic Stewardship Initiative focused on Community-Acquired Bacterial Pneumonia (CABP) in Outpatient Clinics and Urgent Care Centers: A 2023-2024 Community Health System Experience**

Tomefa E. Asempa^1^, Tyler Ackley^2^, Kristin E. Linder^2^, Cara D. Riddle^3^, Eric Walsh^4^, David P. Nicolau^1,5^

^1^Center for Anti-Infective Research and Development, Hartford Hospital, Hartford, CT, USA;

^2^Pharmacy Department, Hartford Hospital, Hartford, CT, USA; ^3^Hartford Healthcare Medical Group, Farmington, CT, USA; ^4^GoHealth Urgent Care, CT, USA; ^5^Division of Infectious Diseases, Hartford Hospital, Hartford, CT, USA

**Supplemental 1**. Guideline concordant therapies used to build order set based on 2019 IDSA/ATS CAP Guidelines and local antibiogram


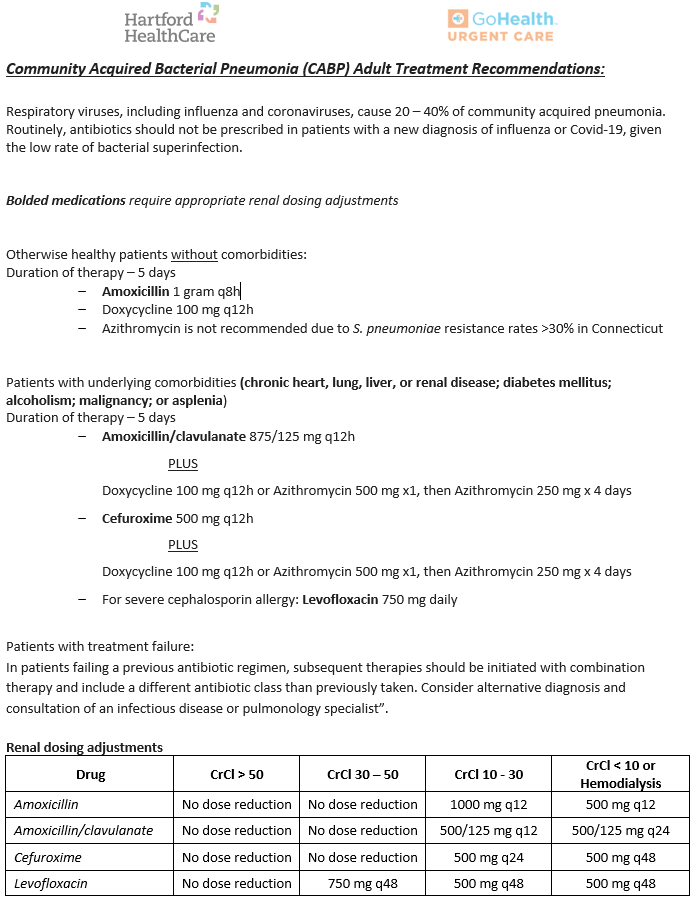


**Supplemental 2**. Community Acquired Bacterial Pneumonia (CABP) EHR Order set Panel Workflow (Tips and Tricks) circulated to all providers as part of awareness campaign.


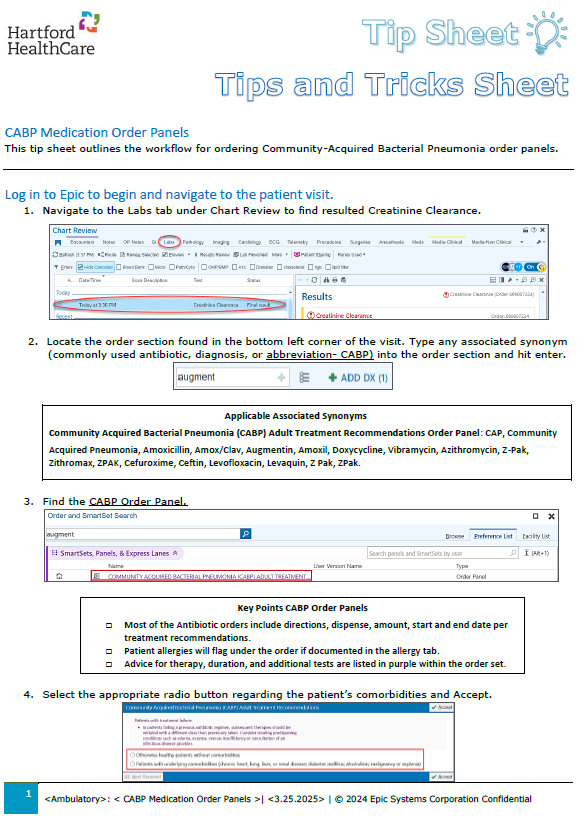


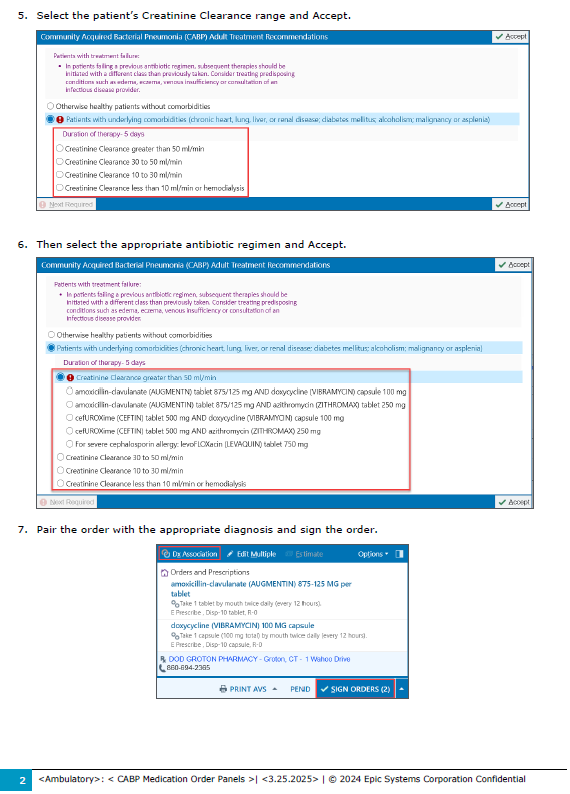


**Supplemental 3**.  List of International Classification of Diseases (ICD)-10 diagnosis codes that were categorized as Community-Acquired Bacterial Pneumonia in the study

A48.1:  Legionnaires' disease

A49.0: Staphylococcal infection, unspecified site

A49.01: Methicillin susceptible Staphylococcus aureus infection, unspecified site

A49.1: Streptococcal infection, unspecified site

A49.3: Mycoplasma infection, unspecified site

B95.0: Streptococcus, group A, as the cause of diseases classified elsewhere

B95.3: Streptococcus pneumoniae as the cause of diseases classified elsewhere

B95.4: Other streptococcus as the cause of diseases classified elsewhere

B95.5: Unspecified streptococcus as the cause of diseases classified elsewhere

B96.0: Mycoplasma pneumoniae [M. pneumoniae] as the cause of diseases classified elsewhere

B96.1: Klebsiella pneumoniae [K. pneumoniae] as the cause of diseases classified elsewhere

B96.3: Hemophilus influenzae [H. influenzae] as the cause of diseases classified elsewhere

J13: Pneumonia due to Streptococcus pneumoniae

J14: Pneumonia due to Hemophilus influenzae

J15: Bacterial pneumonia, not elsewhere classified

J16.0: Pneumonia due to other specified infectious organisms

J18.0: Pneumonia, unspecified organism

J18.1: Lobar pneumonia, unspecified organism

J18.8: Other pneumonia, unspecified organism

J18.9: Pneumonia, unspecified organism
